# Optimal Positioning of Load-Distributing Band CPR Device by Body Mass Index

**DOI:** 10.3390/jcm13175119

**Published:** 2024-08-29

**Authors:** Dong-gyu Kim, Eunhyang Park, Dongsun Choi

**Affiliations:** 1 Department of Emergency Medicine, Seoul Medical Center, Seoul 02053, Republic of Korea; kdg900216@naver.com; 2 Department of Pathology, Severance Hospital, College of Medicine, Yonsei University, Seoul 03722, Republic of Korea; epark54@yuhs.ac

**Keywords:** mechanical CPR device, chest compression, BMI

## Abstract

**Background**: Research investigating the optimal compression position for load-distributing bands (LDBs) in treating cardiac arrest is limited This study aimed to determine the optimal LDB position based on body mass index (BMI). **Methods**: A simulation study was conducted using chest and abdominal computed tomography imaging data collected with patients in the arms-down position. Participants were categorized into three BMI groups: low (<18.5 kg/m^2^), normal (18.5–25 kg/m^2^), and high (≥25 kg/m^2^). The assumed compression area was 20 cm below the axilla. The optimal compression position was identified by adjusting the axilla to maximize the thorax-to-abdomen volume ratio (TAR) and the covered heart volume ratio (CHR), defined as the ratio of heart volume compressed by the LDB to total heart volume. Optimal compression positions were compared across BMI groups. **Results**: Among 117 patients, TAR was significantly lower in the low BMI group compared to the normal and high BMI groups (*p* < 0.001), while CHR differences were not significant (*p* = 0.011). The distance between the optimal position and axilla height was significantly greater in the normal and high BMI groups than in the low BMI group (46.5 cm vs. 66.0 cm vs. 72 cm, respectively; *p* < 0.001). For each unit increase in BMI, the optimal position shifted significantly cephalad relative to axilla height (β coefficient 2.39, adjusted *p* < 0.001). **Conclusions**: Significant differences in TAR were observed among BMI groups. As BMI increased, the optimal LDB position shifted progressively cephalad.

## 1. Background

Cardiac arrest (CA) is a significant public health crisis. Annually, more than 356,000 out-of-hospital CAs occur in the United States [1], with a global survival rate of approximately 8–10% [1,2]. High-quality cardiopulmonary resuscitation (CPR), including effective chest compressions at the correct site, is crucial for survival [3]. Current guidelines recommend compressing the lower half of the sternum based on clinical trials demonstrating improved physiologic parameters [3,4,5]. Imaging studies corroborate this by showing the left ventricle aligns with the lower sternum [6].

Obesity, defined as a body mass index (BMI) ≥ 30 kg/m^2^ is a major cardiovascular risk factor [7,8]. Its prevalence doubled from 1980 to 2015, affecting 12% of the global adult population [9]. Increased abdominal fat displaces the diaphragm upward in obese individuals [10]. Lee et al. found the optimal chest compression point, maximizing left ventricle volume, is higher in obese compared to non-obese patients [11].

Various mechanical CPR devices have been developed [12]. Piston-driven systems align with traditional compression site guidelines based on cardiac pump theory. Conversely, load-distributing band (LDB) devices, operating on thoracic pump principles, compress the entire thorax, rendering previous compression recommendations inapplicable. Current LDBs are positioned at the axilla and compressed downward 20 cm [13]. While used in approximately 30% of mechanical CPR cases [14], optimal LDB placement based on patient body type remains unexplored.

This study aims to determine the optimal LDB position by comparing chest and abdominal volumes compressed by the band across different BMI groups. Using chest and abdominal computed tomography (CT) imaging data, we simulated LDB application and calculated compressed thoracic and abdominal volumes. As LDBs generate cardiac output through increased intrathoracic pressure, we sought to maximize the thorax-to-abdomen volume ratio (TAR) [15]. The optimal compression site was determined by maximizing both thoracic volume relative to the compressed abdomen and compressed heart volume.

## 2. Materials and Methods

### 2.1. Study Design

This retrospective analysis included patients who presented to a single emergency medical center at Seoul Medical Center, located in Seoul, Republic of Korea, between March and July 2021 and underwent simultaneous chest and abdominal CT imaging. Patients were categorized into the following three BMI groups: low (<18.5 kg/m^2^), normal (18.5 to 25 kg/m^2^), and high (≥25 kg/m^2^).

**Figure 1:**
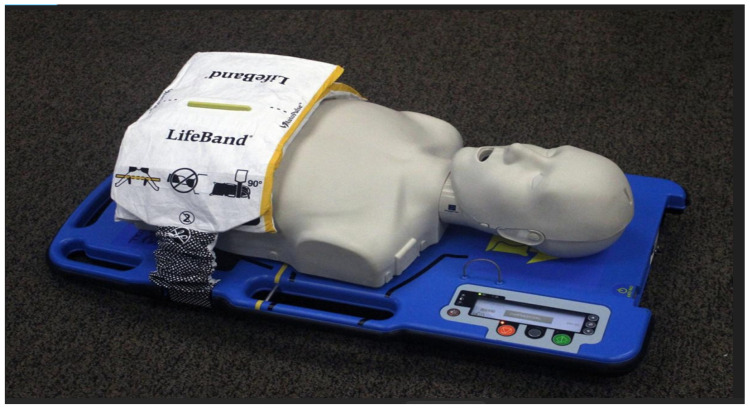
AutoPulse (Zoll Medical Corporation, Chelmsford, MA, USA) applied to a mannequin.

To simulate the AutoPulse (Zoll Medical Corporation, Chelmsford, MA, USA), which is designed to align with the axilla and compress 20 cm below the axilla, patient CT images were used to virtually model thoracic compression from the axilla to 20 cm caudally (Figure 1) [13]. The TAR was calculated as the ratio of compressed intra-thoracic organ volume to the sum of compressed intra-thoracic and intra-abdominal solid organ volumes within this range. The covered heart volume ratio (CHR) represented the proportion of the total heart volume compressed by the simulated LDB.

This study to determine the optimal LDB position based on TAR and CHR, comparing results across BMI groups. This study was approved by the Institutional Review Board of Seoul Medical Center (Seoul, Republic of Korea; Number 2023-09-011-001).

### 2.2. Study Participants

This study included adult patients aged 18 years and older who presented to a single emergency medical center between March and July 2021. Eligible patients underwent contrast-enhanced chest and abdominal CT scans followed by hospital admission. Patients were positioned supine with arms parallel to the torso (i.e., arms-down position) for imaging, and height and weight data are available.

Exclusion criteria encompassed patients with thoracic abnormalities, such as massive pleural effusion, pericardial effusion, severe pneumonia, pneumothorax, or thoracic deformities (e.g., barrel chest). Abdominal conditions, such as solid organ masses and ascites, also led to exclusion. Patients with BMIs below 15 kg/m^2^ or above 35 kg/m^2^, as well as those with thoracic widths between 25 cm and 35 cm, were removed from the study population.

### 2.3. Variables and Outcomes

Patient demographics, including age, sex, height, weight, and BMI, were collected. Patients were categorized into the following three BMI groups: low (<18.5 kg/m^2^), normal (18.5–25 kg/m^2^), and high (>25 kg/m^2^).

**Figure 2:**
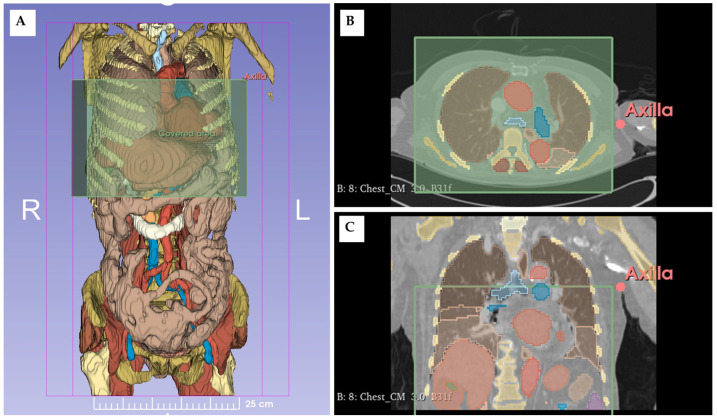
Segmented voxel image of CT scans. (**A**) 3D reconstruction of segmented voxel images showing the band-covered area and armpit level; (**B**) axial view of the CT scan; (**C**) coronal view of the CT scan (pink dot: axilla; green box: band coverage area).

The patient’s axilla was defined as the initial skinfold observed in axial CT images when viewed from cephalad to caudal (Figure 2). Enhanced chest and abdominal CT scans were extracted and analyzed using 3D Slicer version 5.4.0 [16]. The Total Segmentator module in the 3D Slicer segmented each organ within the acquired PACS images (Figure 2) [16]. Segmentation data from chest and abdominal CT scans were merged using T12 (spine) as a reference point.

Data analysis was performed using Python version 3.9 with the ‘pynrrd 1.0.0’ package. Within a 20 cm radius of the axilla, the combined voxel volumes of the trachea, lungs, heart, and pulmonary artery constituted the intra-thoracic organ volume (TV) (Figure 2). The intra-abdominal solid organ volume (AV) included the voxel volumes of the spleen, kidneys, liver, gallbladders, pancreas, and adrenal glands. The TAR was calculated as:

Thorax abdomen volume ratio (TAR) = intra-thoracic organ volume (TV)/(TV + intra-abdominal solid organ volume (AV))

The entire heart volume on the CT image and the heart volume within the 20 cm radius from the axilla determined the CHR:

Covered heart ratio (CHR) = Covered heart volume/Total heart volume

The LDB starting position varied from T1 to T12 spine levels, recalculating TAR and CHR at each position. The optimal compression point maximized TAR while achieving a CHR of 1. The vertical distance between the optimal compression point and the axilla was calculated.

The primary outcomes were TAR and CHR. The secondary outcome was the height difference between the optimal compression point and the axilla.

### 2.4. Statistical Analysis

Continuous variables, including height, weight, organ volume, TAR, and CHR, were summarized using the median and interquartile range. Categorical variables, such as sex and axilla location, were compared across BMI groups using chi-squared tests. The Kruskal–Wallis test evaluated differences in continuous variables among BMI groups. Post-hoc comparisons with Bonferroni correction were conducted, considering *p* < 0.0167 as statistically significant due to multiple comparisons.

The trends in TAR and CHR across different BMI groups were analyzed using mixed models as the LDB starting position varied from T1 to T12. The ‘lme4’ package in R was employed for this analysis, and visualizations were created using the ‘ggplot2’ package.

The optimal LDB position, defined as CHR = 1 and maximized TAR, was compared to the current axilla height using the Kruskal–Wallis test across BMI groups. Multivariable linear regression adjusted for height, age, and sex assessed the relationship between the difference in optimal LDB position and axilla height and BMI. All statistical analyses were performed using R version 4.1.3 (R Foundation for Statistical Computing, Vienna, Austria).

## 3. Results

**Figure 3:**
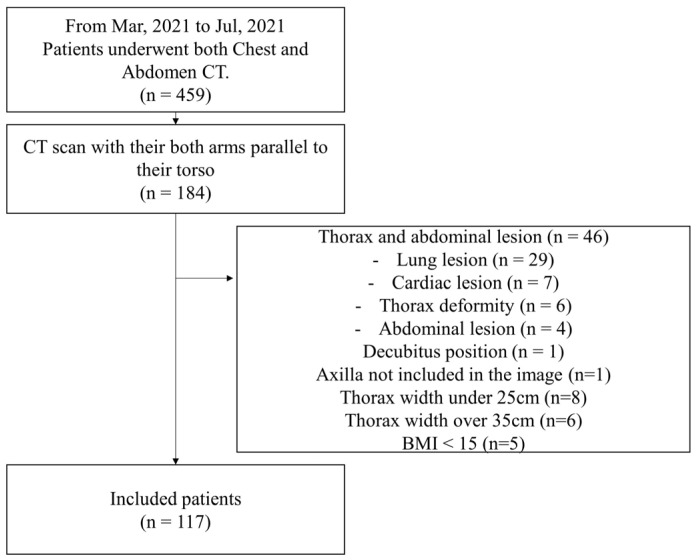
Patient flow diagram.

A flowchart illustrating patient selection is presented in Figure 3. Between March and July 2021, 459 hospitalized patients underwent both chest and abdominal CT scans with available height and weight data. Of these, 184 patients were imaged in the arms-down position. After excluding 46 patients with chest or abdominal lesions, 14 with thoracic widths outside the 25–35 cm range, five with BMI < 15 kg/m^2^, one in the decubitus position, and one without a visible axilla; data from 117 patients were analyzed.

Table 1 summarizes patient characteristics across BMI groups. No significant differences were observed in age, sex, or height among groups. The most common axilla level was T6 in the low BMI group (37.5%) and T5 in the normal and high BMI groups. The median thoracic volume within the LDB range was 2.3 L, without significant group differences. However, median abdomen volume varied significantly across groups: 1.0 L for low BMI, 1.3 L for normal BMI, and 1.5 L for high BMI (*p* < 0.001). Among intra-abdominal organs, liver, spleen, and pancreatic volumes differed significantly between groups. Total and covered heart volumes were significantly larger in the high BMI group compared to both the normal and low BMI groups (covered heart volume, *p* = 0.005; total heart volume, *p* = 0.003). The median TAR for all patients was 0.6, with significant group differences (*p* = 0.001). Although the median overall CHR was 1 without significant group variations, CHR values below 1 were observed in 4.6% of the normal BMI group (3/65) and 20% of the high BMI group (4/20).

**Figure 4:**
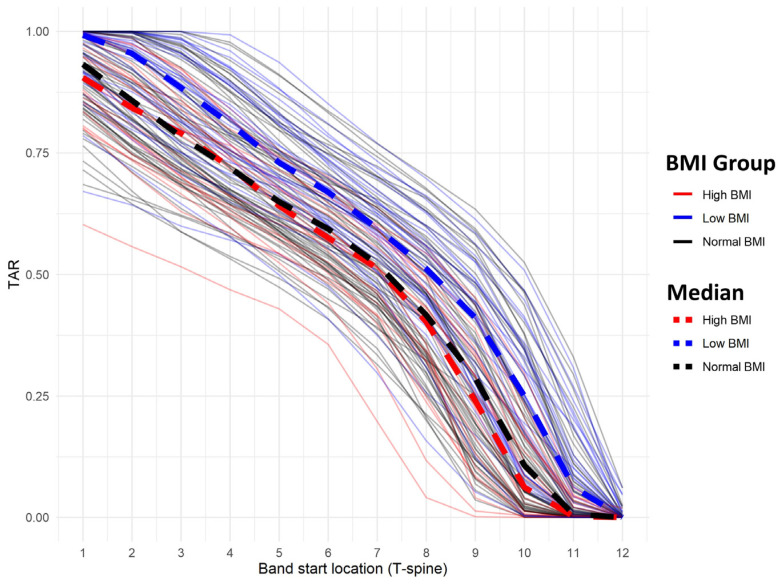
Simulated TAR of each patient according to LDB position and BMI. TAR, thorax abdomen volume ratio; LDB, load-distributing band; BMI, body mass index.

Figure 4 illustrates TAR changes based on LDB starting position across BMI groups. Mixed-model analysis revealed a significant increase in TAR with higher LDB placement (*p* < 0.001). While no significant difference existed between the normal and high BMI groups, TAR differed significantly between the normal and low BMI groups (*p* = 0.0113). The interaction between the BMI group and band location did not significantly influence TAR trends (BMI [low vs. normal) × band location, *p* = 0.62; BMI [high vs. normal] × band location, *p* = 0.78).

**Figure 5:**
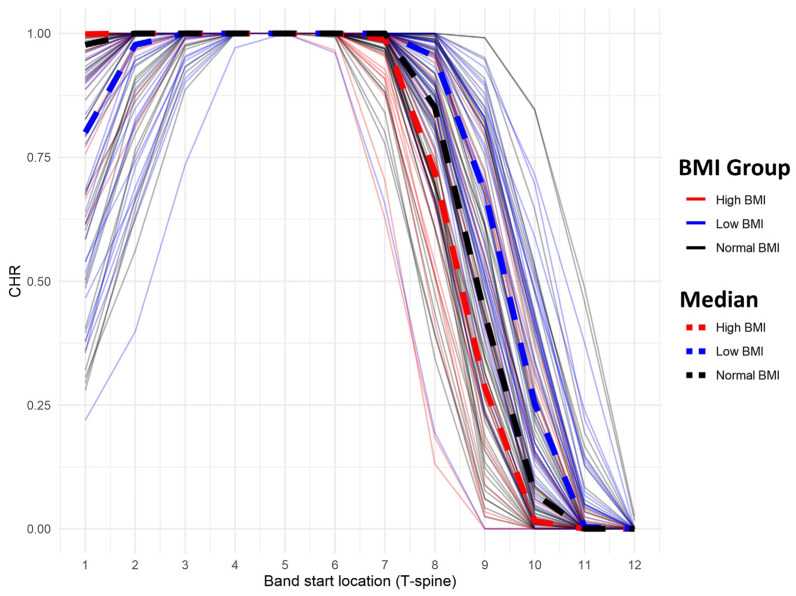
Simulated CHR of each patient according to LDB position and BMI. CHR, covered heart volume ratio; LDB, load-distributing band; BMI, body mass index.

Figure 5 presents CHR changes based on LDB starting position across BMI groups. The LDB level required to achieve CHR = 1 varied by BMI group: T5 for low BMI, T4 and T5 for normal BMI, and T4 and T5 for high BMI. Mixed-model analysis indicated a significant increase in CHR with higher LDB placement (*p* < 0.001). Similar to TAR, CHR differed significantly between normal and low BMI groups (*p* = 0.031) but not between normal and high BMI groups. The interaction between BMI group and band location significantly influenced CHR trends when comparing low and normal BMI groups (*p* < 0.002) but not when comparing high and normal BMI groups (*p* = 0.07).

Table 2 presents a comparison of optimal LDB starting positions across BMI groups. While the mode of the optimal position trended higher in higher BMI groups, no statistically significant differences were observed. The difference between the optimal position and axilla height was significantly greater in the normal and high BMI groups compared to the low BMI group (*p* < 0.001).

Table 3 summarizes the relationship between the difference in optimal position and axilla height relative to BMI. For each unit increase in BMI, the optimal position shifted significantly higher compared to the axilla height (β coefficient 2.39, adjusted *p* < 0.001). Height, age, and sex did not significantly influence this relationship.

## 4. Discussion

### 4.1. Main Findings

This study evaluated the optimal compression position for an LDB based on BMI. To evaluate thoracic compression according to the thoracic pump theory, we simulated the volumes of the thorax, abdomen, and heart compressed by the LDB using CT imaging data. The TAR, representing thoracic compression, was significantly higher in the low BMI group. Conversely, the CHR, indicating direct cardiac compression, did not vary significantly across BMI groups. Notably, the optimal LDB position shifted cephalad with increasing BMI, independent of height.

### 4.2. Imaging-Based Position Optimization for LDB

While imaging-based research on optimal chest compression locations cannot directly measure CPR outcomes, it offers unique advantages. For instance, it allows for focused investigation in specific populations, such as heart failure patients, obese individuals, or children, where clinical CA research is challenging

The “cardiac pump theory” posits that blood flow during CPR is generated by direct heart compression between the sternum and spine as the atrioventricular valves close [17]. Previous studies, aligning with this theory, have identified the maximal heart diameter as the optimal compression point based on cross-sectional CT image analysis of the ventricular area. Conversely, the “thoracic pump theory” proposes that blood flow is driven by changes in intrathoracic pressure rather than direct heart compression [5,6,11,18,19]. This theory underlies the development of devices like AutoPulse (Zoll Medical, Chelmsford, MA, USA) and vest CPR, which increase intrathoracic pressure through broader chest compression [15]. Okuma et al.’s animal study demonstrated that expanding the compression area to increase intrathoracic pressure enhances cardiac output and brain tissue oxygenation [20]. To estimate intrathoracic pressure in our study, we calculated the combined volume of compressed intrathoracic organs, including the lungs and heart. Extra-thoracic pressure was approximated by the volume of compressed intra-abdominal solid organs, with the assumption that a higher TAR indicates a more efficient compression.

### 4.3. Association between BMI and Chest Compression

The relationship between BMI and CPR outcomes remains controversial. While Jain et al. and Ogunnaike et al. reported lower survival rates in underweight patients with in-hospital CA, Wang et al. found poorer outcomes in obese in-hospital CA patients [21,22,23]. These conflicting results may be attributed to BMI’s influence on nutritional status and CPR quality. Wang et al. specifically linked increased BMI and anteroposterior diameter to worse outcomes, suggesting that altered body shape in obese patients negatively impacts CPR efficacy [23].

To optimize chest compressions based on body shape, Lee et al. demonstrated that obesity displaces the diaphragm upward, resulting in a higher left ventricle position in imaging studies [11]. Our study corroborates these findings, showing a higher optimal compression position in individuals with increased BMI, likely due to diaphragm displacement and increased abdominal ratio.

Lee et al.’s simulation study revealed that the standard 5–6 cm compression depth is insufficient to adequately compress the heart (>20%) in obese patients [11]. Obesity-induced diaphragm elevation, combined with increased thoracic cavity and anteroposterior chest diameter, hinders effective chest compression. In contrast to piston-type compressors, the AutoPulse (Zoll Medical, Chelmsford, MA, USA) reduces anteroposterior chest depth by a consistent 20%, potentially mitigating the negative impact of obesity on the size of the thorax [13].

Koga et al.’s observational study reported higher abdominal injury rates with LDB compared to manual CPR in in-hospital CA patients [24]. Our findings of increased abdominal compression in higher BMI individuals support the notion that applying compression at a higher position, as suggested by our study, could potentially reduce these complications in obese patients.

### 4.4. Appropriateness of the Axilla as an LDB Position Marker

The primary objective was to identify the optimal LDB position that maximized thoracic compression while minimizing abdominal compression and ensuring complete heart coverage. Given the heart’s lower thoracic location, ideal placement would involve compressing the lower thorax while avoiding excessive abdominal intrusion. Aligning the LDB’s lower border with the inferior cardiac border could potentially achieve this.

However, current LDB designs, anchored to the axilla and extending downward, may not consistently optimize compressions due to variations in thoracic anatomy and diaphragm position. Utilizing a lower-based anatomical marker instead of the axilla could potentially improve consistency.

Our study found substantial variability in axilla position among patients (T3 to T7) (Table 1), influenced by factors such as arm posture and shoulder height, further emphasizing the limitations of axilla-based positioning. Moreover, the broader compression area of LDBs compared to piston-type devices increased the likelihood of incomplete heart compression, as evidenced by CHR values below 1 in 4.6% (3/65) of patients with normal BMI and 20% (4/20) with high BMI.

To enhance LDB effectiveness, further research is necessary to identify alternative anatomical markers that can reliably optimize chest compression.

### 4.5. Limitations

This study employed CT imaging to stimulate LDB compression, which may not fully capture the complexities of real-world patient physiology. Consequently, clinical validation is essential. Moreover, the device’s positioning behind the patient could induce anatomical shifts, potentially altering compression dynamics. Variations between CT-based and actual axilla positions, coupled with standardized arm abduction posture in CT scans, might have introduced biases, particularly an overrepresentation of lower BMI patients.

This study utilized TAR and CHR as proxies for intrathoracic pressure, assuming that larger compression areas equate to higher pressure. However, this simplification may not accurately reflect the complex physiological changes induced by real-world device applications.

## 5. Conclusions

This study revealed significant differences in TAR across BMI groups, with higher ratios observed in normal and high BMI individuals compared to the low BMI group. Additionally, the optimal LDB position shifted cephalad with increasing BMI, suggesting that a higher placement than the traditional axilla-based approach may be more effective for patients with higher BMIs. The wide variability in axilla position underscores the need for alternative anatomical markers to guide LDB application.

## Figures and Tables

**Table 1:** Variables difference according to BMI group.

	Total	Low BMI	Normal BMI	High BMI	*p*-Value
Variables	(*n* = 117)	(*n* = 32)	(*n* = 65)	(*n* = 20)	
BMI ^1^	20.6 [18.2; 24.1]	17.0 [16.3; 17.9]	21.4 [19.5; 23.4]	26.6 [26.0; 28.2]	
Age	78.0 [67.0; 84.0]	78.0 [68.0; 84.0]	79.0 [68.0; 86.0]	69.0 [60.5; 80.5]	0.107
Sex					0.660
Female	59 (50.4%)	14 (43.8%)	34 (52.3%)	11 (55.0%)	
Male	58 (49.6%)	18 (56.2%)	31 (47.7%)	9 (45.0%)	
Weight (Kg)	53.0 [48.0; 60.0]	44.2 [39.6; 50.0]	55.0 [50.0; 60.0]	70.0 [65.0; 80.0]	<0.001 ^a,b,c^
Height (cm)	160.0 [155.0; 168.0]	159.0 [154.0; 172.0]	160.0 [155.0; 165.0]	160.0 [153.0; 169.5]	0.762
Covered Thorax volume (L)	2.3 [1.8; 3.1]	2.7 [1.9; 3.5]	2.3 [1.8; 2.8]	2.3 [1.8; 2.8]	0.227
Covered Abdomen volume (L)	1.3 [1.0; 1.5]	1.0 [0.8; 1.4]	1.3 [1.1; 1.6]	1.5 [1.3; 1.8]	<0.001 ^a,b,c^
Liver (mL)	1010.3 [803.5;1209.1]	827.1 [669.6;1139.1]	983.3 [836.5;1192.5]	1165.7 [1057.8; 1364.5]	<0.001 ^b,c^
Spleen (mL)	116.4 [72.2; 163.9]	72.3 [46.5; 131.2]	123.2 [82.2; 164.3]	143.8 [96.1; 253.2]	0.001 ^a^
Kidney (mL)	66.7 [22.6; 129.8]	39.7 [16.2; 108.4]	82.1 [23.6; 129.8]	94.4 [50.4; 162.5]	0.127
Pancreas (mL)	29.0 [16.7; 42.3]	17.4 [10.8; 30.6]	30.6 [21.2; 46.2]	38.6 [26.8; 47.4]	0.002 ^a^
Covered heart volume (mL)	404.9 [352.0; 504.3]	387.0 [317.9; 438.1]	406.5 [367.5; 492.1]	507.3 [398.2; 552.0]	0.005
Total heart volume (mL)	405.7 [353.9; 504.3]	387.0 [317.9; 438.1]	406.5 [368.7; 492.1]	507.3 [398.2; 552.0]	0.003 ^b,c^
Axilla position					0.145
T3 level	3 (2.6%)	0 (0.0%)	1 (1.5%)	2 (10.0%)	
T4 level	21 (17.9%)	7 (21.9%)	12 (18.5%)	2 (10.0%)	
T5 level	44 (37.6%)	11 (34.4%)	27 (41.5%)	6 (30.0%)	
T6 level	36 (30.8%)	12 (37.5%)	19 (29.2%)	5 (25.0%)	
T7 level	13 (11.1%)	2 (6.2%)	6 (9.2%)	5 (25.0%)	
TAR ^2^	0.6 [0.6; 0.7]	0.7 [0.6; 0.8]	0.6 [0.6; 0.7]	0.6 [0.6; 0.6]	0.001 ^a^
CHR ^3^	1.0 [1.0; 1.0]	1.0 [1.0; 1.0]	1.0 [1.0; 1.0]	1.0 [1.0; 1.0]	0.011

Data are presented as number (%) or median [interquartile range]. ^1^ Body mass index; ^2^ Thorax-abdomen volume ratio; ^3^ Covered heart ratio; ^a^ *p*-values significant after Bonferroni correction (<0.0167) between low BMI group and normal BMI group; ^b^ *p*-values significant after Bonferroni correction (<0.0167) between low BMI group and high BMI group; ^c^ *p*-values significant after Bonferroni correction (<0.0167) between normal BMI group and high BMI group.

**Table 2:** Comparison of optimal position of the LDB by BMI group.

	Low BMI	Normal BMI	High BMI	*p*-Value
	(*n* = 32)	(*n* = 65)	(*n* = 20)	
Optimal position				0.044
T1 level	4 (12.50%)	19 (29.23%)	10 (50.00%)	
T2 level	8 (25.00%)	24 (36.92%)	6 (30.00%)	
T3 level	10 (31.25%)	10 (15.38%)	2 (10.00%)	
T4 level	9 (28.12%)	12 (18.46%)	2 (10.00%)	
T5 level	1 (3.12%)	0 (0.0%)	0 (0.0%)	
difference from axilla (cm)	46.50 [42.00; 61.50]	66.00 [51.00; 84.00]	72.00 [61.50; 87.00]	0.001 ^a,b^
TAR ^1^	0.89 [0.83; 0.92]	0.86 [0.82; 0.91]	0.85 [0.81; 0.90]	0.427
CHR ^2^	1.00 [1.00; 1.00]	1.00 [1.00; 1.00]	1.00 [1.00; 1.00]	0.277

Data are presented as number (%) or median [interquartile range]. ^1^ Thorax-abdomen volume ratio; ^2^ Covered heart ratio; ^a^ *p*-values significant after Bonferroni correction (<0.0167) between low BMI group and normal BMI group; ^b^ *p*-values significant after Bonferroni correction (<0.0167) between low BMI group and high BMI group.

**Table 3:** Linear regression for the optimal LDB position difference from the axilla according to BMI.

Variable	β Coefficient	95% Confidence Interval	*p*-Value
BMI ^1,^*	2.56	1.50~3.62	<0.001
BMI ^1,^**	2.39	1.31~3.46	<0.001
Height (cm) **	−0.39	−1.06~0.28	0.251
Male vs Female **	−4.49	−16.41~7.41	0.456
Age **	−0.23	−0.56~0.09	0.166

^1^ Body mass index; * univariable linear regression model; ** multivariable linear regression model adjusted for height, age and sex.

## Data Availability

The data underlying this article will be shared on reasonable request to the corresponding author.
